# Activity of docetaxel, carboplatin, and doxorubicin in patient-derived triple-negative breast cancer xenografts

**DOI:** 10.1038/s41598-021-85962-4

**Published:** 2021-03-29

**Authors:** Miguel Martin, Rocio Ramos-Medina, Rebeca Bernat, Jose Angel García-Saenz, Maria del Monte-Millan, Enrique Alvarez, Maria Cebollero, Fernando Moreno, Eva Gonzalez-Haba, Oscar Bueno, Paula Romero, Tatiana Massarrah, Isabel Echavarria, Yolanda Jerez, Blanca Herrero, Ricardo Gonzalez del Val, Nerea Lobato, Patricia Rincon, Maria Isabel Palomero, Ivan Marquez-Rodas, Santiago Lizarraga, Fernando Asensio, Sara Lopez-Tarruella

**Affiliations:** 1 Department of Medical Oncology, Hospital General Universitario Gregorio Marañón Instituto de Investigacion Sanitaria Gregorio Marañon (IiSGM), CIBERONC, Universidad Complutense, Dr Esquerdo 46, 28007 Madrid, Spain; 2 Department of Medical Oncology, Hospital General Universitario Gregorio Marañón Instituto de Investigación Sanitaria Gregorio Marañón, CIBERONC, Madrid, Spain; 3 Department of Medical Oncology, Hospital Clínico San Carlos, CIBERONC, Madrid, Spain; 4 Department of Pathology, Hospital General Universitario Gregorio Marañón, Madrid, Spain; 5 Pharmacy Department, Hospital Universitario Gregorio Marañón, Madrid, Spain; 6 Radiodiagnosis Department, Hospital General Universitario Gregorio Marañón, IiSGM, Madrid, Spain; 7 Gynecology Department, Hospital General Universitario Gregorio Marañón, IiSGM, Universidad Complutense, Madrid, Spain; 8 Experimental Medicine and SurgeryUnit, Instituto de Investigación Sanitaria Gregorio Marañón, Madrid, Spain

**Keywords:** Breast cancer, Cancer models, Cancer

## Abstract

Triple-negative breast cancer (TNBC) is highly responsive to neoadjuvant polychemotherapy regimens including anthracyclines, taxanes, and, more recently, carboplatin. However, there is inadequate information on the individual contribution of each of these agents to the global activity of the combinations, and the use of combinations of up to four of these drugs is associated with relevant toxicity. Identifying single-drug activity in the clinical neoadjuvant setting is challenging. We developed patient-derived xenografts (PDXs) from several chemotherapy-naïve TNBC samples to assess the antitumor activity of single drugs and combinations of drugs. PDXs were established from chemotherapy-naïve TNBC samples. Nine TNBC PDX models (all of which corresponded to a basal-like phenotype according to the PAM50 classifier) were treated with carboplatin, docetaxel, and doxorubicin and the combination of docetaxel and carboplatin. Only one of nine PDX models showed sensitivity to doxorubicin, while eight of nine PDX models showed sensitivity to docetaxel and carboplatin as single agents. The 3 PDX models derived from patients with g*BRCA*-1 or g*PALB2* mutations were very sensitive to carboplatin single agent. All 6 PDX models from patients without hereditary germ-line mutations showed increased sensitivity to the combination of docetaxel and carboplatin. In the present study, docetaxel and carboplatin single agents were active drugs against basal-like TNBC, while doxorubicin monotherapy showed low activity. The combination of docetaxel and carboplatin was more effective than the drugs used as single agents, except in the PDX from patients with *gBRCA1/PALB2* mutations.

## Introduction

Breast cancer is a heterogeneous disease that includes a particular subtype, the triple negative breast cancer (TNBC) subtype, characterized by lack of immunohistochemistry (IHC) staining for estrogen receptor (ER), progesterone receptor (PR), and HER2 receptors^1^. TNBC accounts for approximately 15–17% of all breast cancers and is associated with poorer clinical outcome with regard to the remaining breast cancer subtypes^2^. Approximately three-fourths of all TNBCs belong to the basal-like subtype as defined by the PAM50 gene expression classifier^3^, being usually a highly proliferative and high-grade tumor that more often affects premenopausal women^4^.

No clear druggable targets have been identified in TNBC, and chemotherapy remains the therapeutic backbone of the disease. Local–regional TNBC (UICC stages I–III) is still a curable disease in a significant proportion of patients^2^. Current treatment for local–regional TNBC involves surgery with or without radiation therapy as local therapy and chemotherapy as systemic therapy. Chemotherapy is increasingly used as the initial therapy for local–regional TNBC (neoadjuvant or primary chemotherapy) because pathological response to chemotherapy is a good predictor of patient outcomes in TNBC^5,6^. Classical neoadjuvant chemotherapy for TNBC includes anthracycline-cyclophosphamide-taxane combinations and induces pathological complete responses (pCRs) in breast plus axilla in approximately one-third of patients with TNBC^6^. However, the relative contribution of each chemotherapy agent to the antitumor activity of the combination is unknown. However, carboplatin has been incorporated into neoadjuvant regimens for TNBC to increase the antitumor activity, and this incorporation translates into a higher pCR rate than those obtained using classical regimens^7,8^. All these chemotherapeutic agents have significant toxicity to the combination regimen. The identification of the single-agent activity of taxanes, anthracyclines, and carboplatin would be of great clinical relevance to design rational combination regimens, but neoadjuvant trials focusing this are presently difficult to perform because of the difficulty of funding prospective randomized trials in which new drugs of potential commercial interest are not involved.

We hypothesized that PDX from untreated TNBC could help identify the activity of single chemotherapy drugs and synergism between drugs. We engrafted several chemotherapy-naïve TNBC samples from patients with breast cancer to NOD.Cg-Prkdcscid IL2rgtm1Wjl (NSG) mice to assess the antitumor activity of single drugs and combinations of drugs and correlate the results with the clinical and pathological responses to the drugs in donor patients.

## Results

From 2015 to 2020, a total of 70 tumor core biopsies from therapy-naïve patients with stage II and III TNBC were implanted into the NSG mice. Moreover, 34 of the 70 tumor implants were engrafted (success rate, 49%). All engrafted tumors belonged to the basal-like subtype according to the PAM50 classifier. We selected tumors from nine patients with or without germline *BRCA1* and *PALB2* mutations with different pathological responses to chemotherapy to correlate the results in PDXs with the patient´s responses. In patients with wild-type (WT) *BRCA1/PALB2* (n = 6), two of the selected tumors were obtained from patients with a good pathological response after neoadjuvant chemotherapy (Symmans class 0 or I), two from patients who, despite having a partial clinical response, had extensive residual disease (Symmans class III), and the remaining two from patients with good partial clinical response and moderate residual disease (Symmans class II). The same experiment was performed in all 9 PDX models. Five groups of treatment with ten mice in each group were analyzed. Among PDXs obtained from patients with *BRCA1/PALB2* germline mutations, two showed a good pathological response to neoadjuvant chemotherapy (class 0), and the last one had moderate residual disease (Symmans class II). Patient and tumor characteristics are presented in Table 1. When the relative change in KI67 expression was evaluated as a measurement of the quantification of cell proliferation, a relevant decrease was observed after docetaxel and carboplatin as single agents as well as after their combination.

**Table 1:** Baseline characteristics of patients and clinical response to the docetaxel plus carboplatin regimen.

Patient and disease feature from the diagnostic core	*N* = 9 (%)
**Age (year)**	
˂50	4 (45%)
≥ 50	5 (55%)
**Menopausal status**	
Pre	4 (45%)
Post	5 (55%)
**Clinical T-stage**	
T1	1 (11%)
T2	5 (55%)
T3	2 (23%)
T4	1 (11%)
**Clinical N-stage**	
N0	2 (23%)
N1	6 (66%)
N2	1 (11%)
N3	0 (0%)
**Symmans class**	
pCR	3 (33%)
RCB-I	1 (11%)
RCB-II	3 (33%)
RCB-III	2 (23%)
**Clinical response (MRI)**	
Complete response	2 (23%)
Minor response	5 (55%)
Partial response	1 (11%)
Progression disease	0 (0%)
Stable disease	1 (11%)
**Ki 67 expression**	
˂50	1 (11)
≥ 50	8 (89%)
**Histologic grade**	
G1	0 (0%)
G2	2 (23%)
G3	6 (64%)
No data	1 (11%)
**Germline homologous recombination mutation**	
BRCA wt	6 (66%)
BRCA mut	2 (22%)
PALB2 mut	1 (11%)
**Current status (the last follow-up date)**	
Recurrence and death	2 (22%)
Recurrence and alive	1 (11%)
Alive and no recurrence	6 (66%)

*MRI* magnetic resonance imaging, *pCR* pathological clinical response, *RCB* residual cell burden, *T* tumor, *N* nodes, *G* grade.

### Histopathologic and genomic characteristics of tumors

Histopathologic characterization of the nine PDX models showed concordance with the corresponding patient’s tumor in terms of lack of expression of hormonal receptors (ER, PR) and absence of HER2 amplification (data not shown). In situ hybridization with an Alu probe specific to human cells showed the human origin of all engrafted tumors. Next-generation sequencing (NGS) of somatic DNA confirmed that the germline *BRCA1* and *PALB2* mutations harbored by some patients were also present in the corresponding PDX.

### Response to anticancer drugs in PDXs

**Figure 1:**
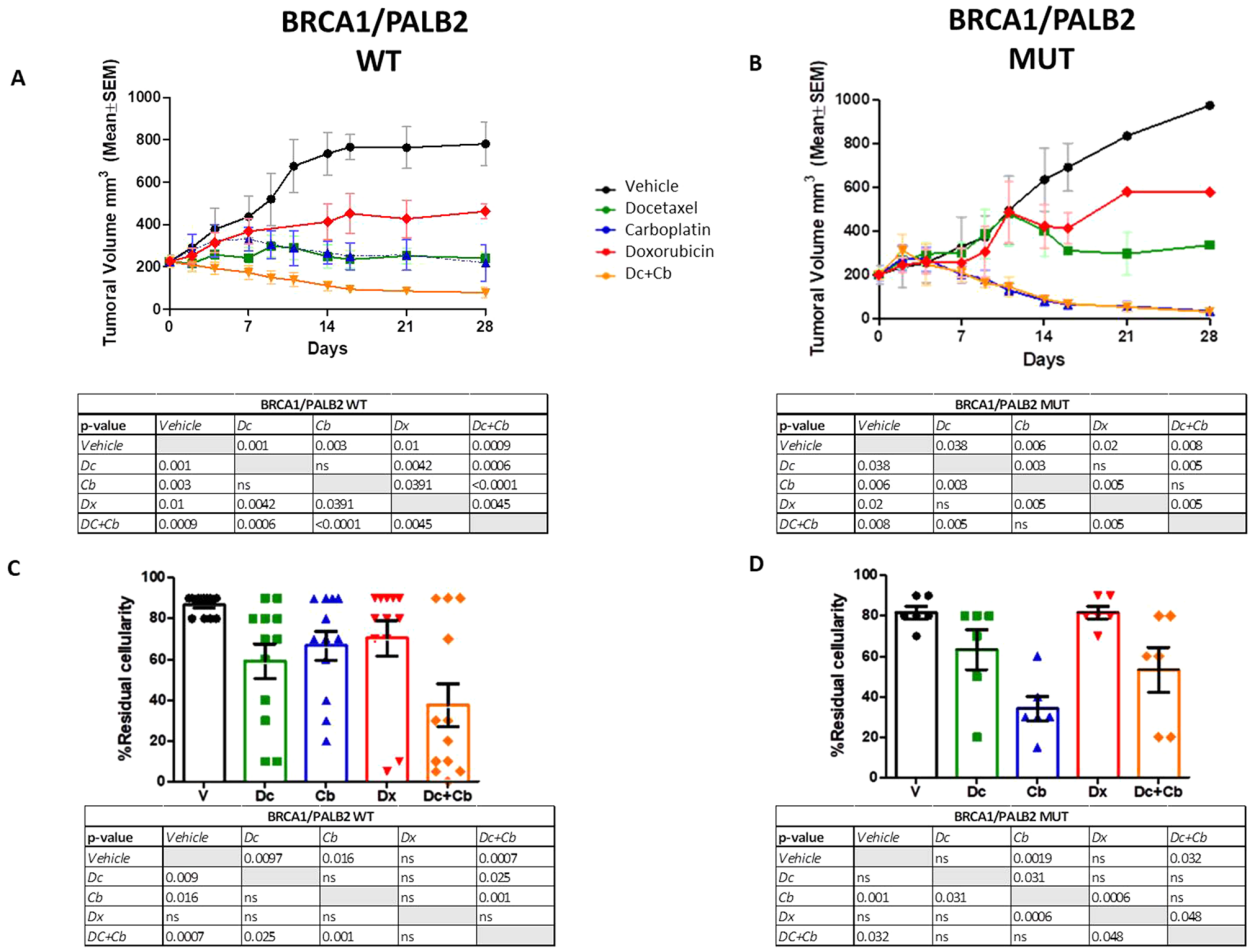
Tumor volume evolution (panels A and B) and residual cellularity (panels C and D) in PDX from patients with and without mutations in genes involved in homologous recombination. ns, nonsignificant; *p < 0.05; **p < 0.005; ***p < 0.0005; Dc, docetaxel; Cb, carboplatin; V, vehicle; Dx, doxorubicin; Dc + Cb: docetaxel plus carboplatin.

Figure 1 shows the aggregated evolution of the volume of PDX (images A and B) and its residual cellularity (images C and D) in response to anticancer drugs and according to *BRCA1/PALB2* status (WT vs. mutated). In WT tumors, docetaxel and carboplatin showed more antitumor activity than doxorubicin; the combination of docetaxel and carboplatin showed greater antitumor activity, with a median tumor growth inhibition (TGI) of 95% (*p* < 0.0019). In *BRCA1/PALB2* mutated tumors, carboplatin was the most active drug in reducing tumor volume. The median TGI of carboplatin in this subgroup was 97% (*p* < 0.0001). In these tumors, adding docetaxel to carboplatin did not improve the antitumor activity.

In terms of residual cellularity after treatment, the combination of docetaxel and carboplatin led to the greatest reduction in PDX from patients without *BRCA1/PALB2* mutations (62%, *p* = 0.0007). The reductions in cellularity were also significant with carboplatin (*p* = 0.016) and docetaxel (*p* = 0.0097) but not with doxorubicin. PDXs from *BRCA1/2* and *PALB2* germline mutation carriers showed significant reduction in cellularity with carboplatin (*p* = 0.0019) and with the combination of carboplatin and docetaxel (*p* = 0.032).

**Figure 2:**
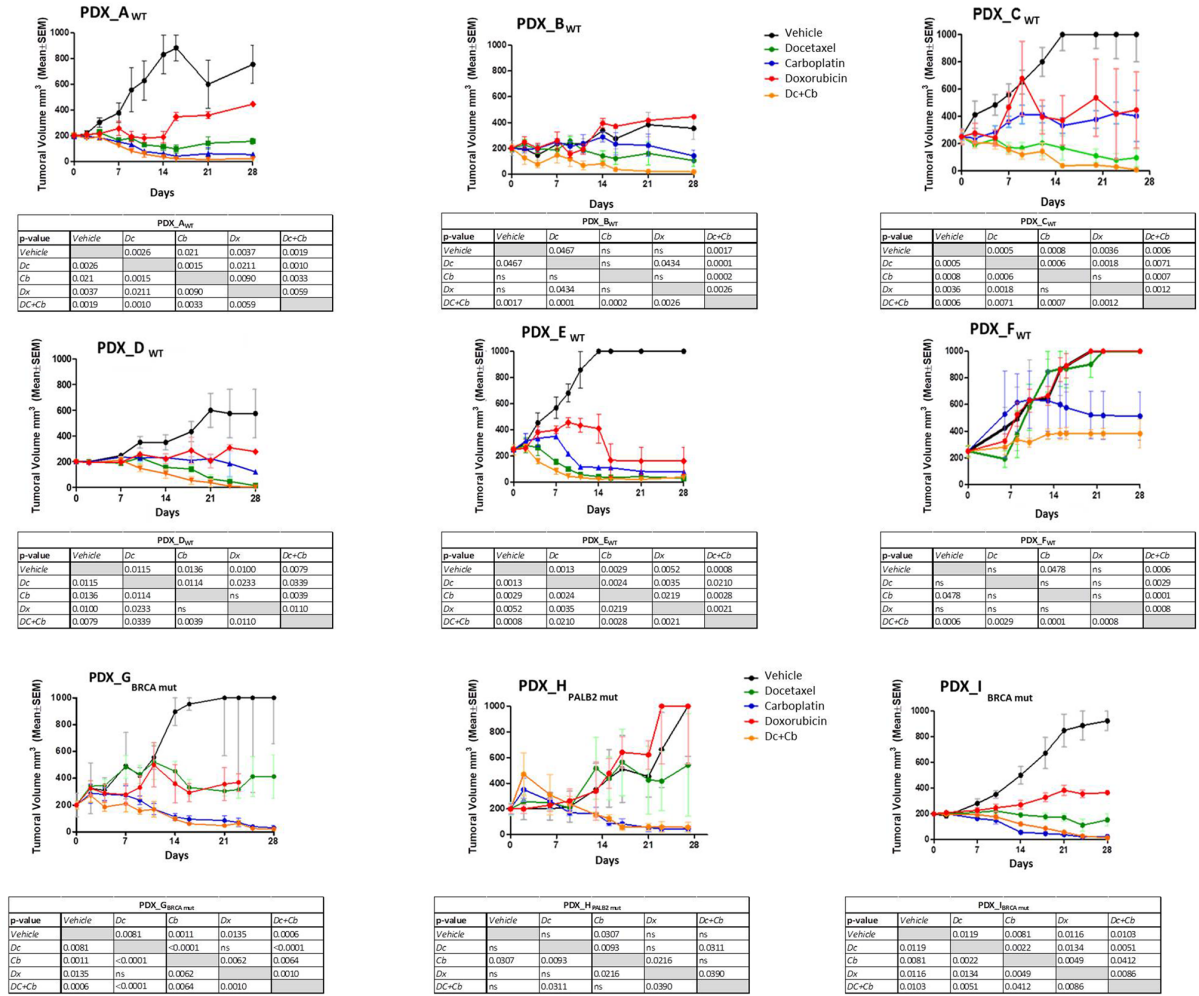
Tumor volume evolution. Volume reduction in the nine PDX models with ten mice per group WT, BRCA1/PALB2 WT; MUT, BRCA1/PALB2 mutation; PDX, patient-derived tumor xenograft; NAC, neoadjuvant chemotherapy; ypTNM iatrogenic pathological TNM after NAC treatment; RCB, residual cancer burden; Dc + Cb, docetaxel + carboplatin; TV, tumor volume; TGI, tumor growth inhibition; D0, Day 0; D28, Day 28. Tumor volume was measured using a caliper three times a week and calculated as [tumor length × tumor width^2^]/2.

Figure 2 shows the individual results of the nine PDX models with ten mice in each therapy group. Eight showed low or null sensitivity to doxorubicin. Only one (PDX-E) showed sensitivity to this drug. Eight of nine models showed good sensitivity to docetaxel and carboplatin as individual agents, but the combination regimen achieved the best antitumor response except in PDX from patients with *gBRCA1/PALB2* mutations.

### Correlation between response in PDXs and patients

PDXs from patients with excellent (A, B, G, H) or moderate (C, I) response to neoadjuvant chemotherapy also showed an excellent response to docetaxel plus carboplatin. The median follow-up period for patients who were alive was 36 months. Patients with excellent responses were alive without recurrence for 23 months (patient A), 58 months (patient B), and 60 months (patients G and H). Patients C and I were alive without relapse after 57 months and 22 months, respectively.

One of the two PDXs (F) obtained from patients with large residual disease after neoadjuvant chemotherapy (Symmans class III) showed minimal sensitivity to docetaxel plus carboplatin. However, the remaining PDX (E) from a patient with a clinical partial response but poor pathological response (Symmans class III) was sensitive to docetaxel plus carboplatin in the mouse models. Patients F and E died after 22 and 24 months of follow-up, respectively.

Overall, there was a good correlation between clinical and PDX responses (8/9 models, 89%).

### Changes in Ki67 expression

**Figure 3:**
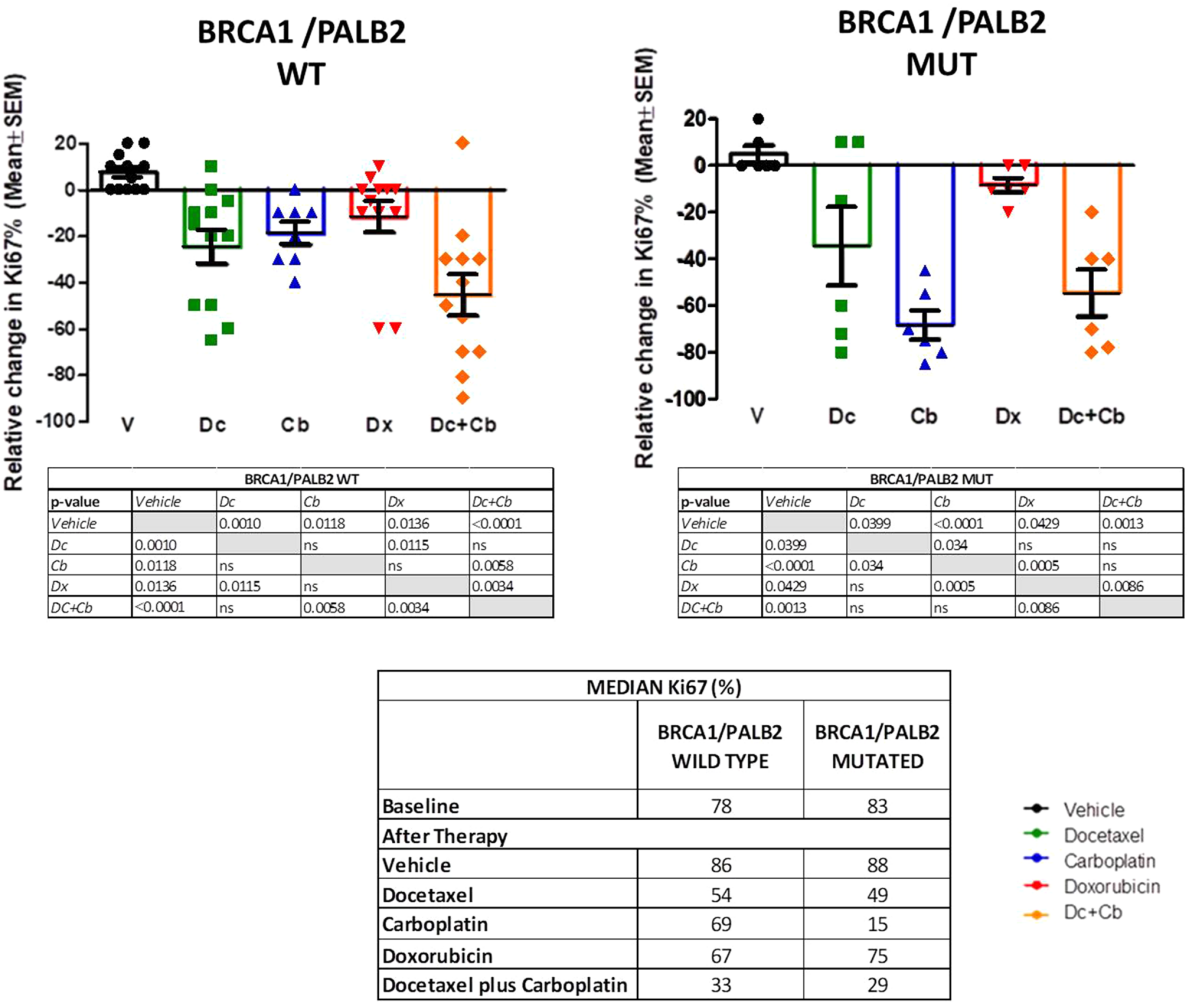
Ki67 changes after therapy in PDX. The reactions were carried out in an automated immunostaining platform (Autostainer Link AS48, Agilent). The slides were incubated with mouse monoclonal anti-KI-67 (MIB-1, Ready to use, Agilent, IR626). After the primary antibody, slides were incubated with the visualization systems (EnVision FLEX + Rabbit or Mouse linker, Dako) conjugated with horseradish peroxidase. Immunohistochemical reaction was developed using 3, 30-diaminobenzidine tetrahydrochloride (DAB) including in FLEX Kit and nuclei were counterstained with Carazzi’s hematoxylin. Finally, the slides were dehydrated, cleared and mounted with a permanent mounting medium for microscopic evaluation. ns, nonsignificant; *p < 0.05; **p < 0.005; ***p < 0.0005; Dc, docetaxel; Cb, carboplatin; V, vehicle; Dx, doxorubicin; Dc + Cb, docetaxel + carboplatin.

The expression of the proliferation marker Ki67 was measured in representative tumors from each of the treatment groups and PDXs models before and after therapy. All tumors showed a high Ki67 level before therapy (> 70% of stained cells). In PDX derived from patients with *BRCA1/PALB2* WT tumors, the KI67 expression after therapy was significantly lower in the groups treated with docetaxel (*p* = 0.0006) and D + Cb (*p* = 0.0006). In PDX derived from patients with *BRCA1/PALB2* mutations, carboplatin (*p* =  < 0.0001) and D + Cb (*p* = 0.0013) produced a significant reduction in Ki67 (Fig. 3).

### Molecular characterization of PDXs

PAM50 breast cancer intrinsic subtypes were analyzed in 144 formalin-fixed paraffin-embedded xenograft samples (three samples per treatment group) using the nCounter platform. A basal-like subtype was confirmed in all samples.

### Next-generation sequencing

After sequencing analysis, we observed the same mutation profile between germinal DNA from patient and somatic DNA from PDX. Likewise, when we compare the mutations before and after the treatment, we didn´t observe any difference.

### Evaluation of TOP2a

Three independent observers analyzed the FISH results of the nine PDXs models to search for TOP2a copy number alteration. None of the samples showed amplification of the TOP2a gene (Fig. 1 supplementary).

## Discussion

We established several PDXs using tumor samples from patients with triple negative breast cancers who were naïve to any therapy, in order to have a model allowing for testing chemotherapy drugs commonly used as neoadjuvant therapy for this disease (docetaxel, carboplatin, doxorubicin). Orthotopic breast tumorgrafts retain critical characteristics of the original tumor specimens from breast cancer patients, including the tumoral and the stromal components^9^ and can, therefore, help to test the efficacy of a antitumor drug or combinations.

All engrafted TNBC xenografts we obtained were of basal-like subtype as defined by the PAM50 classifier and, therefore, the findings of our study can only by applicable to this intrinsic subtype of breast cancer (around 70% of all TNBC)^3^.

Our study found a somewhat different pattern of sensitivity to drugs in PDX coming from chemotherapy-naïve TNBC patients with and without mutations in genes involved in DNA homologous recombination repair (*BCRA1* and *PALB2*) and hereditary breast cancer. Some studies that evaluate the efficacy of carboplatin as an individual agent or as a combination show a vast improvement in TNBC tumors with *BRCA* mutations. In one of the studies, athymic mice were inoculated intra-cerebrally with a *BRCA*-mutant cell line (basal and claudin-low) or *BRCA* WT cell line. They proved that carboplatin + /− the PARP inhibitor ABT888 in murine intracranial (IC) TNBC models significantly improved survival in *BRCA*-mutant IC models compared to control but did not improve survival in *BRCA* WT IC models. Moreover, they saw that carboplatin + ABT888 revealed a modest survival advantage versus carboplatin in *BRCA*-mutated models. Our data continues to support the role of carboplatin for the treatment of breast cancer, specifically in TNBC with *BRCA* mutations^10,11^. In our study, PDX coming from patients without mutations in *BRCA1/PALB2* were sensitive to both docetaxel and carboplatin and, particularly to the combination. On the other hand, PDXs from patients with germline mutations in *BRCA1/PALB2* were extraordinarily sensitive to single agent carboplatin and moderately sensitive to docetaxel. Outstandingly, doxorubicin was the less effective single drug regardless of *BRCA1/PALB2* gene status.

The assessment of antitumor activity in our study was based on the evaluation of tumor volume shrinkage, residual cellularity and changes on Ki67. All the results were concordant with respect to sensitivity to the tested drugs.

Our study, therefore, suggest that docetaxel and carboplatin (and its combination) are active treatments in PDXs derived from basal-like TNBC breast cancer tumors, while doxorubicin is clearly less active.

Taxanes (docetaxel and paclitaxel) are an integral part of all combination chemotherapy regimens for early TNBC, since its addition to classical regimens has clearly improved the outcome of the patients^9,12–14^. In PDX from patients without g*BRCA1/PALB2* mutations, our study supports the interest of the combination of docetaxel plus carboplatin. Conversely, in PDX from patients with g*BRCA1/PALB2* mutations, carboplatin is the most active single drug and docetaxel seems to be of limited value, as suggested in other studies^15^.

Our findings that docetaxel alone or in combination is effective in TNBC PDXs from patients without *gBRCA1/PALB2* mutations (by large, the most frequent subgroup of TNBC breast cancer) are coincident with other PDX literature studies. A Korean group studied the antitumor activity of docetaxel, bevacizumab, and its combination. The results of the study show that the treatment with either docetaxel or bevacizumab significantly decreased the tumor volumes of PDX generated from TNBC tumors after receiving breast-conserving surgery and adjuvant chemotherapy^16^.

The role of carboplatin in the treatment of TNBC is still a matter of debate. A recent meta-analysis of randomized trials comparing neoadjuvant regimens with or without carboplatin showed that the inclusion of the drug in the neoadjuvant regimen was associated with a significant increase in the pathological complete response rate, a surrogate of good survival outcome. Two of the studies included in the meta-analysis reported overall survival (OS). They found a relative reduction in mortality with carboplatin of 14% that was not statistically significant. (HR 0.86, 95% CI 0.46–1.63, *P* = 0.651)^17^. Tumors arising in patients bearing a germline mutation in genes involved in homologous recombination (i.e. *BRCA 1* and *2*, *PALB2* and others) seems to have a different sensitivity to chemotherapy drugs than their non-mutated counterparts, being particularly sensitive to DNA damaging drugs. The TNT trial compared docetaxel to carboplatin in patients with metastatic TNBC and/or germline *BRCA* mutations. In non-mutated patients, both drugs were similarly active while in *BRCA* mutated tumors carboplatin was significantly more active than docetaxel. Interestingly, there was no complete cross-resistance between docetaxel and carboplatin, suggesting the practical interest of combining both drugs, as we did in our study. Our experiment showed that most basal-like TNBC PDXs were sensitive to carboplatin (particularly, those with *BRCA1* or *PALB2* mutations), supporting the efficacy of the drug seen in clinical trials.

Although the data previously published regarding the anti-tumor effect of doxorubicin in TNBC PDXs models are not directly comparable to ours. However, Evans et al.^18^. made a study with five PDX models from residual disease after chemotherapy and two PDX from treatment-naïve tumors. Their results showed six PDX progressed on doxorubicin and one chemo-naïve tumor, presented stable disease but not response. Doxorubicin is considered a key drug in the (neo) adjuvant therapy of breast cancer, since many trials and a comprehensive meta-analysis showed that anthracycline-containing combinations were superior to the prior standard, the CMF regimen (cyclophosphamide, methotrexate, fluorouracil) in unselected populations of breast cancer patients^19^. A meta-analysis of trials comparing CMF vs anthracycline combinations in studies in which the HER2 status of the tumors was available found that the benefit of anthracyclines was limited to HER2-positive tumors^20^. A subsequent meta-analysis with similar aim showed again a greater benefit with anthracyclines versus CMF in patients with HER2-positive tumors (hazard ratio for relapse of 0.70, 95% CI 0.51–0.96). Patients with HER2-negative tumors also have a much smaller benefit. In the triple negative population, the hazard ratio for relapse was 0.80 (non-significant)^21^. The selective activity of anthracyclines in HER2-amplified breast cancer could be related to the simultaneous amplification of the Top2a gene, the main target of anthracyclines, placed in the same amplicon as the *HER2/neu* gene. Copy number alterations of the TOP2a gene occur almost exclusively in association with *HER2/neu* amplification^22^. In our experiment, none of the PDX had topo2a copy number alterations and this could explain the low activity found with doxorubicin. In the clinical setting, a randomized neoadjuvant phase II trial, comparing 4 cycles of full dose doxorubicin (75 mg/m^2^ every 3 weeks) to 4 cycles of full dose docetaxel (100 mg/m^2^ every three weeks), showed that docetaxel was significantly more active than doxorubicin in the specific subgroup of patients whose tumors were of the basal-like subtype. In this subtype, docetaxel-treated patients have a significantly higher rate of good pathological responses (Symmans class 0 + 1, 56% vs 0%, p = 0.029) and a significantly lower residual cancer burden (1.626 ± 0.499 vs 3.245 ± 0.483, *p* = 0.039) than doxorubicin^23^.

The role of anthracyclines as adjuvant therapy of early breast cancer has been object of further debate since the introduction of taxanes (docetaxel and paclitaxel). These drugs were first added to anthracycline-containing combinations. In several adjuvant randomized trials, the addition of a taxane to the anthracycline combination, either in sequence or in combination, produced a significant reduction in the risk of relapse as compared with the non-taxane arm^24,25^. Since anthracyclines can have irreversible side-effects (i.e. cardiac toxicity, acute leukemias) Jones et al. designed an anthracycline-free regimen, the combination of docetaxel and cyclophosphamide (TC). In an adjuvant phase III randomized trial, these investigators showed that 4 cycles of TC were superior to 4 cycles of AC (doxorubicin plus cyclophosphamide) in terms of disease-free survival and overall survival, both in the overall population and in the TNBC population^14,26^. A German trial, the PLAN B study, compared 6 cycles of TC with 4 cycles of anthracyclines followed in sequence by 4 cycles of docetaxel as adjuvant therapy for patients with HER2-negative breast cancer tumors. Both regimens were of identical efficacy, including in the subpopulation of TNBC patients^27^.Conversely, a combined analysis of three different adjuvant trials including more than 4000 patients, comparing 6 cycles of the TC regimen to standard taxane-doxorubicin-cyclophosphamide (TaxAC) regimens, found a small but statistically significant benefit with the doxorubicin-containing regimens (4-year IDFS of 88.2% and 90.7% for TC and TaxAC, respectively, *P* = 0.04)^28^. In a recent study, the combination of carboplatin plus taxanes was of similar efficacy to epirubicin plus cyclophosphamide followed by taxanes as adjuvant chemotherapy for early triple‑negative breast cancer. In the small proportion of patients with germline BRCA1 mutations, the carboplatin regimen was associated with less recurrences than the anthracycline regimen^29^. In a neoadjuvant randomized phase II study, the combination of docetaxel and carboplatin had the same proportion of pCR as that of multidrug regimen including anthracyclines, taxanes, cyclophosphamide, and carboplatin^30^.

In our experiment, docetaxel and carboplatin were effective drugs in TNBC PDXs. Conversely, these PDX showed a limited sensitivity to doxorubicin. These results are concordant with the clinical experience discussed before and suggest that taxanes and carboplatin (but not doxorubicin) are crucial drugs in TNBC, although doxorubicin can have some activity in a limited proportion of patients.

The main strength of our experiment is the use of therapy-naïve TNBC specimens that mimics the scenario of neoadjuvant therapy in women with TNBC, and the inclusion of a significant number of mice in each group. Our experiment has some limitations as well. The lack of PDXs coming from non-basal TNBC (around 25% of TNBC according to literature^3^ prevents to generalize the results to the whole population of TNBC.

The correlation between the results found in the PDXs with the response of the tumor in the real life was good in eight out of nine patients (89% of cases). The remaining patient has a large tumor with axillary involvement that obtained a partial reduction with docetaxel plus carboplatin (from 58 to 26 mm in diameter), but the residual tumor burden after chemotherapy was high (class III due to the presence of 4 axillary lymph nodes involved with metastases.

## Conclusion

In the present study, docetaxel and carboplatin single agents were active drugs, while doxorubicin showed limited activity, in PDX from patients without gBRCA1/PALB2 mutations and TNBC. The combination of docetaxel and carboplatin was more effective than the drugs used as single agents. In PDX from patients with gBRCA1/PALB2 mutations, carboplatin was the most active agent.

There was a good correlation between the clinical response to docetaxel and carboplatin in patients and the response of PDX to the same combination in eight of the nine models.

## Methods

### Patients

Human breast cancer core biopsies were collected from newly diagnosed TNBC patients (chemotherapy naïve) with UICC stage II or III disease, from two institutions (Hospital Gregorio Marañón and Hospital Clínico San Carlos, Madrid, Spain).The sample collection was approved by the corresponding ethics committee (collection identifier: C.0000188 at ISCIII, Spain) and all patients provided written informed consent. All methods were performed in accordance with the relevant guidelines and regulations, in accordance with the Declaration of Helsinki.

Patients whose tumors were implanted into mice were enrolled into the trial with registration number NCT01560663(22/03/2012) and treated with neoadjuvant docetaxel (75 mg/m2 i.v.) plus carboplatin (area under curve 6 i.v., every 3 weeks) for 6 cycles prior to surgery. The results of this clinical study (NCT 01560663) have been previously published^31^. Clinical response was assessed by magnetic resonance imaging of the breast, prior and after chemotherapy. Pathological responses in surgical specimens were classified according to the residual cell burden (RCB) system by Symmans et al.^32^. The combination of docetaxel and carboplatin was highly active in women, achieving a 55% of RCB class 0 responses (complete clearance of the tumor from breast and axilla -pCR-) and a 13% of class I responses (minimal microscopic residual disease in breast without axillary residual tumor). Both classes of pathological responses are associated with a similar, excellent, survival outcome^33,34^.

### Ethics approval and consent to participate

The human study protocol was approved by Comité de Ética de la Investigación con Medicamentos (CEIm), Hospital General Universitario Gregorio Marañón. The IiSGM Animal Care and Use Committee and Comunidad de Madrid approved all the PDX procedures (PROEX 137/16). Procedures involving animal care complied with national and international laws and policies. The collection of samples was approved by the corresponding ethics committee (collection identifier: C.0000188 at ISCIII, Spain). Patients provided written informed consent before any procedure.

### Mice and establishment of tumor xenografts

NOD.Cg-Prkdcscid IL2rgtm1Wjl (NSG) mice (female, 8-week-old) were obtained from Jackson Laboratories, Bar Harbor, ME, USA. The mice were maintained under specific pathogen-free conditions in the animal facility of one of the institutions (IiSGM). The mice were fed and provided with autoclaved water ad libitum.

**Figure 4:**
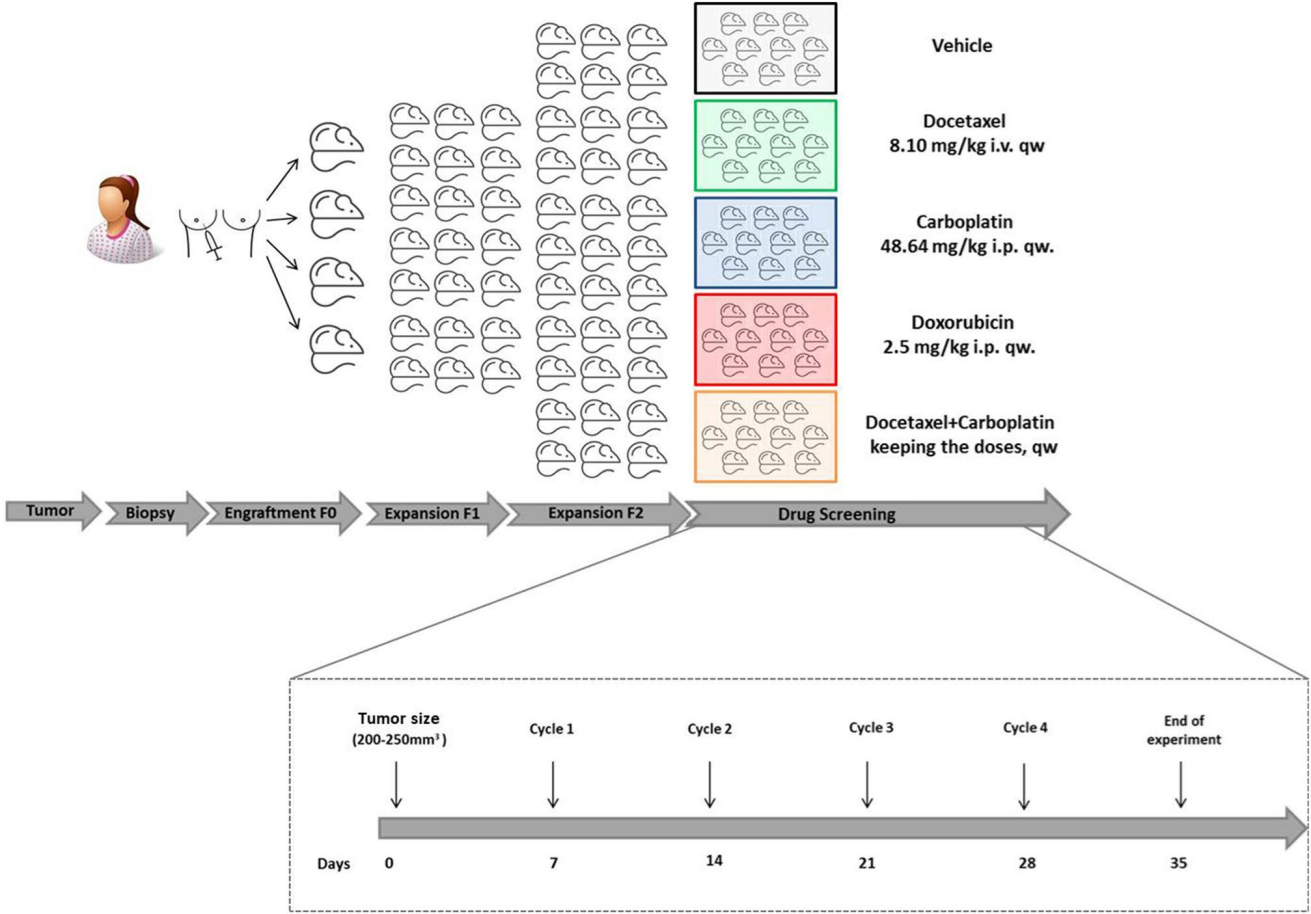
Experimental design of the trial. F0, first engraftment; F1, expansion cohort; F2, treatment cohort.

Tumors were implanted into the 4^th^inguinal mammary fat pads (orthotopically) by two small incisions in the skin to expose the fat pad: one along the midline and one a short distance down the leg, to make a pocket into the center of the transplantation site. Tumor growth was monitored once per week by palpation and caliper measurement. Once the first tumor (named F0 in future references) reached an estimated volume of 1000 mm^3^, it was removed for the expansion process (Fig. 4).

### Confirmation of the nature of TNBC xenografts

To confirm the human nature of implanted the tumors, an Alu probe in situ hybridization was carried out in all the 9 xenografts after engraftment (F0).

Serial 4-µm-thick sections were cut from each paraffin-embedded PDX tumor. Examinations of tumor morphology were conducted on slices processed for H&E staining.

The triple negative breast cancer status of the xenografts was established by immunohistochemistry.The slides were incubated with the appropriate primary antibody as detailed: rabbit monoclonal anti-Estrogen Receptor Alpha (EP1, Ready to use, Agilent, IR084), mouse monoclonal anti-Progesterone Receptor (PgR636, 1/500, Agilent, M3569), rabbit polyclonal anti-c-erBb-2 (1/400, Agilent, A0485) and mouse monoclonal anti-KI-67 (MIB-1, Ready to use, Agilent, IR626). All these determinations were performed before and after treatment.

### PDX treatment protocol

The same experiment was performed in all 9 PDX models. Xenografts from the second mouse to mouse passage (F2) were allowed to grow to an estimated volume of 200-250mm^3^, at which time mice were allocated into the following five groups of treatment with ten mice in each group: (a) vehicle (ethanol, 20.8 mg/ml, 200ul); (b) Docetaxel 8.10 mg/kg i.v once a week^35,36^; (c) Carboplatin 48.64 mg/kg i.p once a week;^10^ (d) combination docetaxel + carboplatin 48.64 mg/kg + 8.10 mg/kg i.v + i.p once a week; (e) Doxorubicin2.5 mg/kg i.p once a week^37^. Mice were treated for 28 days, monitored daily for signs of toxicity, and were weighed three times a week. (Fig. 4).

### Evaluation of response to drugs in PDX

Tumor size was evaluated three times per week by caliper measurements. Tumor volume was calculated using the following formula: Tumor Volume = [tumor length x tumor width^2^]/2.

Tumor growth inhibition (%TGI) was calculated as (1 − (Average relative tumor volume treatment group/Average relativetumor volume vehicle group) * 100).

Experiments were terminated on day 35. The proportion of residual tumor cells and the volume of the residual tumors were measured for the 5 therapeutic groups of each xenograft.

All the experiments in the study were performed in compliance with current ARRIVE guidelines^38^.

### Molecular characterization of breast cancer patient-derived xenografts

#### Extraction RNA

The invasive area of the tumor from haematoxilin and eosin stained slides was delimited by a pathologist at the LAOT and was subsequently microdissected in 10 μm slices. RNA was then extracted using the FFPET RNA Isolation Kit (Qiagen). RNA concentration and quality were assessed with Nanodrop 2000 Spectophotometer (Thermo Scientific NanoDrop Products) according to A260/280 ratio, which needs to be around 2.00 for RNA.

#### Extraction DNA from fresh tumour

DNA was extracted from fresh tumour sample of PDX using phenol/chloroform/isoamyl method. DNA concentration and quality were assessed withNanodrop 2000 Spectophotometer (Thermo Scientific NanoDrop Products), according guidelines, concentration needs to be around 50 ng/ul.

#### Intrisic subtype classification

We analyzed the expression of the 50 genes included in the PAM50 assay and 5 additional housekeeper genes as described by Parker et al.^39^. In the raw nCounter (NanoString Technologies, INC Seattle, WA) transcript quantification the background was corrected using the negative probes and normalizing with their mean minus 2 standard deviations and those values were normalized by calculating the geometric mean of the 5 housekeeper genes. Subtype classification was based on the nearest of the 5 centroids.

#### *Fluorescence *in situ* hybridization (FISH) of topoisomerase II alpha (TOP2A)*

TOP2A amplification was measured by FISH (Vysis-Abbot). The probe used was a locus-specific identifier TOPO2A on 17q21-22, labeled in orange and the centromere (CEP17) was hybridized with spectrum green on 17p11.1-q11.1 (Vysis-Abbott). Sections 3 µm thick were cut from each paraffin-embedded tumoral piece extracted from the 9 PDXs without treatment. All procedures for the FISH analysis were prepared according to the manufacturer’s instructions. Images were analyzed on a fluorescence microscope. TOP2A was determined in a minimum of 20 nuclei counted per case. A positive result was defined as TOP2A:CEP17 ratio of 2 or greater^22^. A tumor was considered to have deleted TOP2A if the ratio was 0.8 or lower^21^.

### Sequencing analysis of PDX

Sequencing libraries were created using 25 ng of genomic DNA from 45 tumor samples belonging to different experimental groups. The panel includes 111 genes exons of which 12 are specifically studied for hereditary breast cancer *(BRCA1, BRCA2, PALB2, RAD50, RAD51C, RAD51D, BARD1, ATM, BRIP1, CHEK2, NBN, PTEN)*. The library preparation, target capture and sequencing were carried out in the genomic systems unit of the Gregorio Marañon Health Research Institute (IiSGM). The sequencing was performed using the MiSeq platform (Illumina), according to the manufacturer’s instructions.

### Variant calling

For variant calling we focused on single nucleotide substitutions, small insertions and deletions (indels). The list of variants was filtered by rare variants (MAF < 0.01) (I), variants in coding exons and splicing junctions (II), discarding variants predicted to produce synonymous amino acid changes without splicing alteration (we only selected high/medium impact variants: frameshift indels and non-sense, missense and canonical splice site variants) (III). Next, we discarded benign and probably benign classified variants (IV) based on different databases (CLINVAR: https://www.ncbi.nlm.nih.gov/clinvar/; Leiden open variation database: http://www.lovd.nl/3.0/home;—BRCA exchange: http://brcaexchange.org/; Locus specific databases: http://grenada.lumc.nl/LSDB_list/lsdbs; varsome (https://varsome.com/); Pubmed: https://www.ncbi.nlm.nih.gov/pubmed/). Variants were classified according ACMG guidelines. A minimum depth of 30% is required for the posterior validation analysis of pathogenic variants with Sanger Sequencing using the Integrative Genomics Viewer (IGV).

### Data analysis

Statistical analysis and graphical presentations were performed using GraphPad Prism 5 and Microsoft Excel. Descriptive data were generally expressed as mean ± standard deviation or standard error of the mean. Statistical evaluations of thedifferences between groups were assessed using student’s t-test analyses, with aprespecified alpha of 0.05.

## Supplementary Information

Supplementary Information 1.

Supplementary Information 2.

Supplementary Information 3.

Supplementary Information 4.
