# Supplementary figures and images for: Activity of docetaxel, carboplatin, and doxorubicin in patient-derived triple-negative breast cancer xenografts
Source: Sci Rep. 2021 Mar 29;11:7064. doi: 10.1038/s41598-021-85962-4 (PMC8007714; doi:10.1038/s41598-021-85962-4)

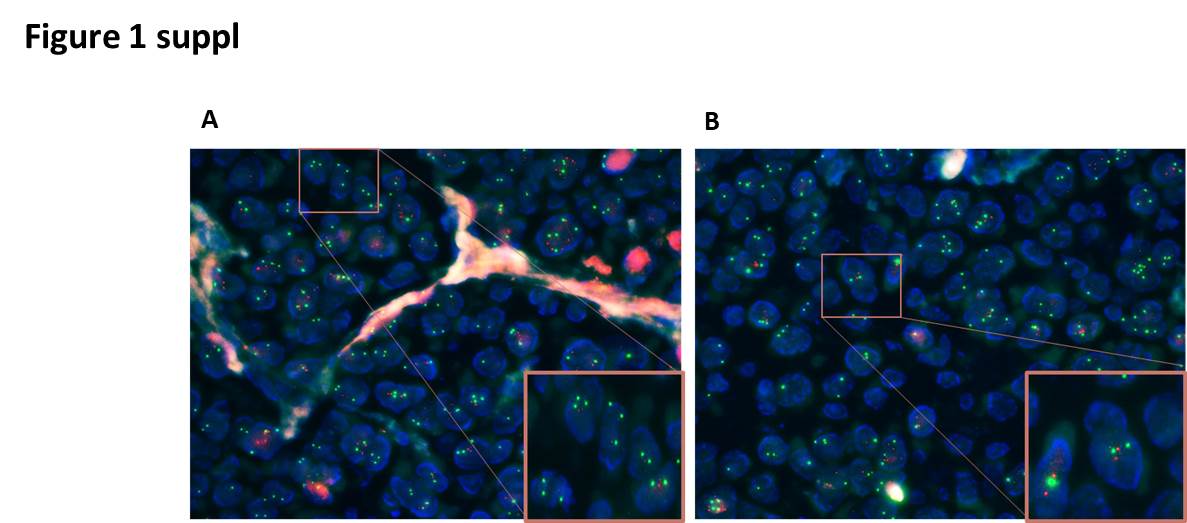

Supplement: Supplementary file 1 — Supplementary Information 1. [file 41598_2021_85962_MOESM1_ESM.jpg]

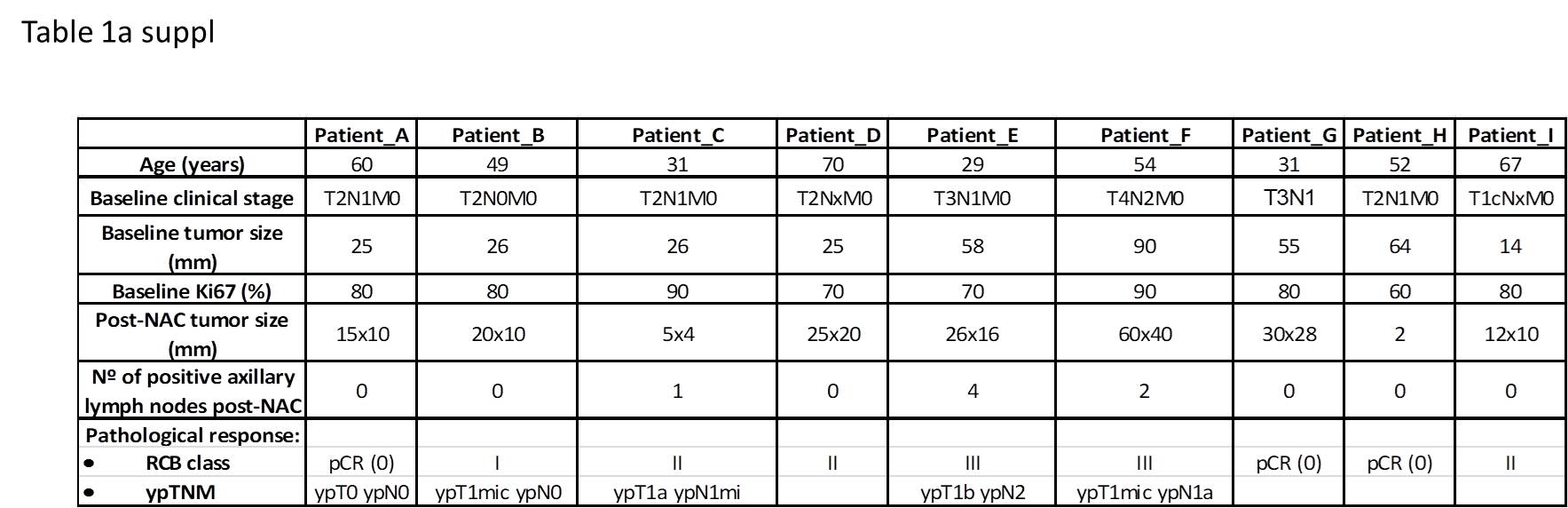

Supplement: Supplementary file 3 — Supplementary Information 3. [file 41598_2021_85962_MOESM3_ESM.jpg]

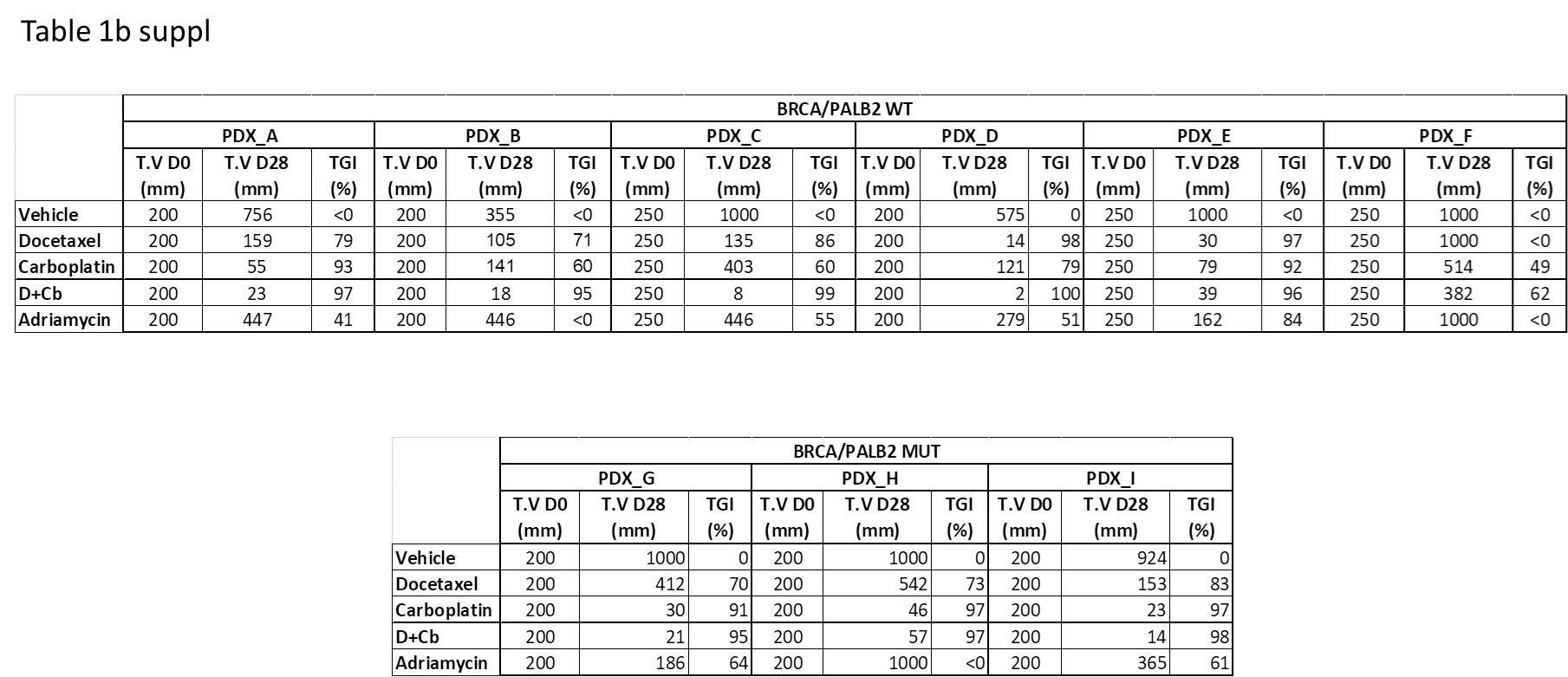

Supplement: Supplementary file 4 — Supplementary Information 4. [file 41598_2021_85962_MOESM4_ESM.jpg]
