# Supplementary material for: Activity of docetaxel, carboplatin, and doxorubicin in patient-derived triple-negative breast cancer xenografts
Source: Sci Rep. 2021 Mar 29;11:7064. doi: 10.1038/s41598-021-85962-4 (PMC8007714; doi:10.1038/s41598-021-85962-4)
Supplement: Supplementary file 2 — Supplementary Information 2. [file 41598_2021_85962_MOESM2_ESM.doc]

**Table and figures legends Supplementary material**

**Figure 1 Supplementary.**

Representative FISH images from two PDX models (TOP2A/CEP17 probe). A) PDXs tumoral cells showing deletion of the TOP2A gene. B) PDX tumoral cells with normal status of the TOP2A gene. Coloration: TOP2A: red, CEP17: green.

**Table 1 Supplementary.**

A) Clinical Information of patients. B) Tumor growth inhibition % (TGI) was calculated as (1-(average relative tumor volume treatment group/average relative tumor volume vehicle group) *100).
